# Assessment of fishes, sediment and water from some inland rivers across the six geopolitical zones in Nigeria for microplastics

**DOI:** 10.5620/eaht.2024018

**Published:** 2024-06-18

**Authors:** Victoria Funmilayo Doherty, Idowu Ayisat Aneyo, Oluwatoyin Tirenioluwa Fatunsin, Christian Ebere Enyoh, Tajudeen Olanrewaju Yahaya, Ikechi Godslove Emeronye, Oluwatosin Aishat Amolegbe, Nnamdi Henry Amaeze, Felix Emeka Anyiam, Aderonke Ajibola Oloidi, Folashade Ajagbe, Oluwaseun Popoola, Moses Ugochukwu

**Affiliations:** 1 Department of Biological Science, Yaba College of Technology, Yaba, Lagos, Nigeria; 2 Department of Zoology, Faculty of Science University of Lagos, Akoka, Yaba, Lagos Nigeria; 3 Department of Chemistry, Faculty of Science University of Lagos, Akoka, Yaba, Lagos Nigeria; 4 Graduate School of Science and Engineering, Saitama University, Japan; 5 Department of Biological Sciences, Federal University, Birnin Kebbi, Kebbi State, Nigeria; 6 School of the Environment, University of Windsor, Windsor, Ontario, Canada; 7 Centre for Health & Development, University of Port-Harcourt, Rivers State, Nigeria; 8 Department of Science Laboratory Technology, Yaba College of Technology, Yaba, Lagos, Nigeria

**Keywords:** Abundance, Environmental contamination, Inland rivers, Microplastics, Nigeria, Pollution

## Abstract

In Nigeria, limited research has been conducted on Microplastics (MPs) in inland rivers, necessitating a comprehensive assessment to understand the extent of contamination. This study aimed to assess the abundance, distribution, and composition of MPs in fishes, sediment, and water from inland rivers across Nigeria's six geopolitical zones. Samples were collected from selected rivers in each geopolitical zone (Rivers Yauri, Benue, Argungu, Jamare, Ogun, Ethiope and Orashi). MPs were isolated using a combination of filtration, density separation, and visual identification. MPs abundance, distribution, shapes, colors, and chemical composition were determined using microscopy and Fourier-transform infrared spectroscopy. The study presents the first report of MPs in six in land rivers in Nigeria and found that MPs were present in all the fishes, sediments and river waters studied across all the rivers. The abundance and composition of MPs varied among the different sample types, with fibers being the most abundant shape in both water and fish samples. PET, PP, and PE were the most prevalent types of plastics found in fish samples, while PE/PA/Nylon, PVA, and PVC were predominant in water samples. PA/Nylon, PUR, PVC, and PET were the most common in sediment samples. Source analysis by Principal component analysis (PCA) and Hierarchical Cluster Analysis (HCA) indicated that the presence of MPs was mainly influenced by local anthropogenic activities. However, estimated daily intakes are generally low, indicating that daily consumption of the samples is not likely to be harmful. The widespread presence of MPs in inland rivers across Nigeria highlights the urgent need for effective waste management strategies and environmental conservation efforts to mitigate plastic pollution.

## Introduction

The interactions of environmental factors in the presence of mechanical abrasion with biological processes influence the breaking down of larger plastics to form microplastics (MPs) [1]. MP pollution has become a crucial environmental issue worldwide, and Nigeria is not left out of this problem [2]. According to Enyoh et al. [3], additional inland rivers and their inhabiting organisms in Nigeria should be examined for macro and microplastic pollution for better understanding of the distribution and prevalence in Nigeria's water bodies. The problem of microplastics in Nigeria's water has given rise to other multitude of challenges with potential consequences for both the environment and human health [4]. With the insufficient number of thorough investigations and monitoring initiatives on MPs pollution in Nigeria's water, the extent of pollution is unknown [5]. Environmental matrices, such as water samples, sediments, and aquatic organisms including fishes, provide representative samples of the ecosystem, and helps to decipher the presence, distribution, and abundance of MPs in different aquatic environments accurately [6] [7]. The exposure pathway of MPs in the food chain poses a risk to many lower invertebrate and vertebrate species that mistakenly pick up these tiny plastics as food in water and sediment. This poses great risk of entanglement, injury and ingestion to biodiversity [8]. The organisms become a reservoir for MPs, which eventually biomagnify across the food chain [9] eventually contaminating the fishes and through the food chains these contaminates and are transferred to humans once consumed, posing a danger to human health.

In the study by Maulana et al., [10] on the Krueng Aceh River in Banda Aceh City, Indonesia, two fish species normally caught for food were caught and assessed for MPs. The two species are mullet *Mugil cephalus* and bagok catfish *Hexanematichthys sago*. Seven to eight MPs per fish was observed for bagok catfish *Hexanematichthys sago* samples from both the river estuary and residential areas, while lower MPs count were associated with agricultural areas. For the mullet *Mugil cephalus*, the highest number of microplastic particles were found in fishes caught in the river estuary (16 particles/fish on average), followed by the sample from residential areas (10 particles/fish on average) 5 particles per fish were recorded in samples from agriculture area. The fiber MPs were predominant in their study and based on the peak values, polyethylene and polypropylene were predominant. Dada and Bellow [11] analysed MPs in 30 fish samples of various species in the Lagos Lagoon, and MPs were found in the stomachs of three carnivorous fish species (*H. odoe*, *C. nigrodigitatus*, and *L. maximus*) but not in the herbivorous *O. niloticus*. Aina et al. [12], assessed eight species of fishes for MPs. Thirty-eight *Coptodon zillii*, fourty-three *Oreochromis niloticus*, nineteen *Sarotheron melanotheron*, three *Chrysicthys nigrodigitatus*, three *Lates niloticus*, one *Paranchanna obscura*, one *Hemichromis fasiatus*, and one *Hepsetus odoe* (a total of 109 fishes) were sampled between February and April 2018 from Eleyele Lake, Eleyele catchment area in Ido local government area, Ibadan, Oyo State Southwest-Nigeria. MPs were observed in all the species except *H. fasciatus*. The size of MPs found in their samples varied from 50 µ m to 5 mm. The study focused on various size fractions as reported by Sani et al. [13], who found MPs in the gastrointestinal tract, gills and muscle tissues of thirty *Oreochromis niloticus* and thirty *Tilapia zilli* collected from Sallari and Hauren Shanu burrow pits of Kano Nigeria, in between April to June 2021. Mps in form of fibers, fragments, microbeads and tyre-dusts were found in all the tissues of the two species examined. Mahu et al. [14] also assessed the gastro-intestinal tracts (guts) of 160 fishes from Nigerian Coastal waters immediately after landing by purchasing them at Makoko community seafood market in Lagos, Nigeria and recovered a total of 5744 MP with an average of 39.65 ± 5.67 items/individual. The MPs recovered were microbeads, fragments, burnt film, thread, fibers and pellets in nature. The size of MPs found in their samples varied from 124 micrometers (µ m) to 1.53 millimeters (mm). MPs found in *Coptodon zillii*, *Oreochromis niloticus* and *Sarotherodon melanotheron* ranged from 124 µ m to 1.5 mm while for other species, such as *Paranchanna obscura*, *Chrysicthys nigrodigitatus*, *Lates niloticus*, and *Hepsetus odoe*, it ranged from 1 mm to 1.53 mm. They associated the sizes of MPs found in the various fishes with habitat, feeding habits, and trophic levels of the fish. Idowu et al. [15], assessed MP pollution and silver catfish (*Chrysichthys nigrodigitatus*) in water from Osun River system, Nigeria and found abundant number of MPs in their sample (maximum of 22,079 ± 134 particles/litre for water and a range of 407 ± 244 to 1691.7 ± 443 particles for fishes). Their report recorded the highest reported Microplastic concentration so far for a river water globally.

Ololade et al. [16], assessed five inland rivers in south western Nigeria for MP namely Ibese River in Lagos State, Ogbese River in Ondo State, Ona River in Oyo State, Asejire River, in Oyo State, Ogun River in Ogun State and one coastal ocean water- the Third Mainland Bridge Ocean in Lagos State. They reported abundant amount of MP in sediments and water samples across all locations ranging from 12.82 to 22.90 particle/kg dw and 6.71 to 17.12 particle/L (dry season) and 5.69 to 14.38 particle/kg dw and 12.41 to 22.73 particle/L (wet season), respectively. Foam and fiber were the lowest and highest type of particules respectively in all their sediment and water samples while polypropylene and polyethylene were the prevalent MPs materials.

Ogbomida et al. [17], assessed MPs in water, sediment, and fish species (*Clarias gariepinus* and *Oreochromis niloticus*) from the Ikpoba River, Edo South Eastern Nigeria. They observed polypropylene, polystyrene and polyethylene were prevalent in the surface water, polyvinyl chloride and polyethylene terephthalate were prevalent in the sediment samples while polyethylene was prevalent in the fishes. Fiber, film, foam, were common in all the sample types. The *C. gariepinus* accumulated higher concentration of MPs compared to *O. niloticus* fish. The size of the MPs in water, sediment and fish samples ranged from 50 µ m to 5 mm, 100 µ m to 5 mm and from 124 µ m to 1.53 mm respectively with highest concentration associated with primary consumers. There is a dearth of information on MPs on fishes, sediments and river water from inland rivers in Nigeria. Most of the previous studies on MPs in fishes, rivers and sediments were on the coastal water that are at the external boundaries of Nigeria [11], [14] the other study on MPs in fishes in Nigeria was on fishes from a lake [12] and a burrow [13]. The few studies on inland waters were limited to some southern water bodies in Nigeria. There is no study on MPs in inland water from the northern part of Nigeria and there are no studies on Rivers Ethiope and Orashi in the southern part of Nigeria. Plastic waste are being discharged into the aquatic systems in Nigeria due to poor management practices. Plastic production and consumption stood at 436 kilotons and 1,090 kilotons ‘in 2018 respectively, causing increase in the abundance of plastics in the inland freshwater system, estimated to be about 283,293.47 hectares, of which 70% has been degraded due to the pollution [3]. This study presents the first assessment of MPs in fishes, rivers and sediments from six inland rivers (namely River Yauri, River Benue, River Argungu, River Jamare, River Ethiope and River Orashi ) in Nigeria. This research aims to develop evidence-based data about the presence, abundance, and types of MPs in fishes from seven inland freshwaters in six geo-political zones of Nigeria, and to determine the risk associated with it towards developing an appropriate policy and management framework for sustainable water quality.

## Materials and Methods

### Study area

**Figure 1.**
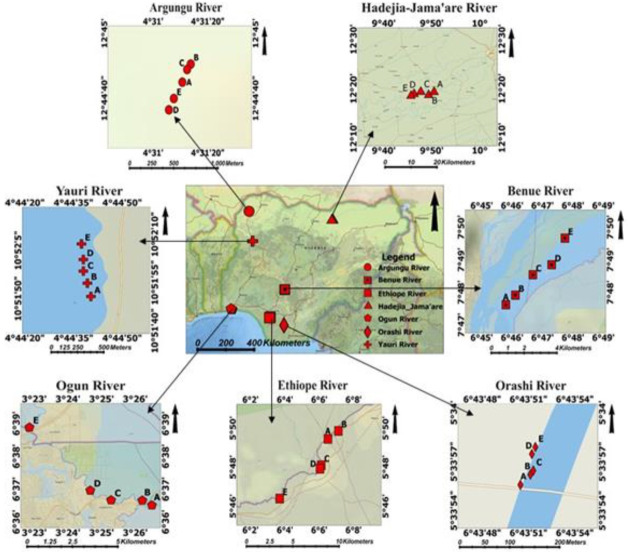
Map of the study Areas in Nigeria.

The samples were obtained from seven rivers in Nigeria, as shown in the map (Figure 1). They are River Yauri (which is part of the River Niger passing through Yauri town), River Benue, River Argungu, River Jamare (all in the northern part of the country), River Ogun, River Ethiope and River Orashi (all in the southern part of Nigeria). The River Niger (River Yauri) and its drainage basin cover 2,117,700 km2 and extend 4,180 km. The river links Nigeria to the Republic of Guinea through Mali and Niger and flows southeast through a vast, shallow valley of 8 to 16 km wide downstream from Jebba, Nigeria. The significant tributary Kaduna River joins it around 110 km from Jebba, contributing about one-fourth of the river's annual volume below the Niger-Kaduna confluence. The river bends south 40 km above Lokoja to meet the River Benue, which is the River Niger's largest tributary and supplies water at Lokoja. The River Benue is wider at its confluence. They form a two-mile lake with islands and sandbanks. Yauri is a local government area in Kebbi State, northwestern Nigeria, and home to one of Nigeria's smallest medieval emirates [19] Niger River basin covers 7.5 % of the African continent and spreads over ten countries, including Nigeria. River Argungu is in Argungun town in Kebbi State, Nigeria. Farming tobacco, peanuts, rice, millet, wheat, sorghum rice, millet, sorghum, cotton, cow, goat, and sheep are the majour occupations. The town is known for its annual fishing festival. The river, provides food and irrigation for their farms. The Jama'are (Jamare) River, also known as the Bunga River in its upper sections, begins in the highlands near Jos, Plateau State, Nigeria, and runs northeast through Bauchi and Yobe States before joining the Hadejia River to form the Yobe River. The Hadejia-Jama'are River Basin is a Sahelian biodiversity reservoir in Nigeria. Hadejia-Nguru wetlands, a Sahel, are important bird habitats fed by the Hadejia and Jama'are rivers. The Hadejia River Basin spans 11°32′08.4″N to 12°26′24.8″N and 8°07′50.0 [19].

River Ogun is one of the rivers in the south-western part of Nigeria covers a total area of about 22.4 km2. It transverses through Ibarapa, Iseyin, Abeokuta, Owode, Ikorodu, and Ifo local government areas before finally discharging into the Lagos Lagoon. This site has become a place of interest considering its constant and continuous pollution, owing to the fact that it serves as a focal point of some commercial activities. River Ethiope in Abraka, Delta State, is a fast-flowing river with a rate of approximately 0.80 - 1.0 m/s. The River is greenish, crystal clear, and saltless. It was the main drinking water source for the communities along its bank but presently, it is mainly used for recreation such as swimming and picnicking. One other major activity is dredging of the crystal white sand from it used for civil construction of building and roads. This dredging activities have led to dispersed fine sand particles in the water resulting in high total suspended solids of the river, less in-water visiblility and reduced crystal-like appearance. Usually, another water body called Orime River empties into the Ethiope river changing the crystal green colour to dull green and reduces the pH. The Orashi River (also called Urashi or Ulasi River) is in the lower basin of the Niger River and a tributary of Lake Oguta in southeastern Nigeria. The river is in Egbema, Imo State, Nigeria and is 250 km long and 183 m above sea level [20] The Orashi River serves as a drinking, washing and other multi-purpose river for communities such as South Ideato community, Mmahu and Abacheke in Egbema Imo State. It flows through Ogba and Ahoada in Rivers State, Urualla, Akokwa, Okija, Orsu, Ukpor, Ihiala, Uli, Oguta, Osemotor, Omoku, Obiakpor, Ebocha, Ukodu, Okarki, Mbiama and Epiare in Imo State from Dikenafai. This river forms tributaries along its route from Imo to Anambra, Rivers to Bayelsa before flowing to Atlantic. The Orashi river is referred to as “an ecosystem of national importance” due to its significance for local Communities in sustaining livelihood, maintaining biodiversity, providing clean water and recreation [20].

### Sampling

Fishes were caught from five sites in the seven rivers by fishermen and preserved with 70 % ethanol. Water samples and corresponding sediments were also obtained at the sampling points. The water samples and soil samples were made into composites and preserved samples were transported the same day to arrive the next day in the laboratory for analysis. The seven rivers were sampled between February and March, 2023 (dry Season, 2023).

### Extraction of microplastics in fish

The body weight (g) and total length (cm) of each fish were recorded before dissection. The gastrointestinal tract (GIT) of each fish, was weighed and digested using 10 % potassium hydroxide at 40 °C for 12 hours [21], [22]. The digested liquids were then filtered through a 20 µ m pore size cellulose membrane filter of 47 mm diameter (Millipore, NY2004700) using a Buchner funnel and a pump. The membrane was then placed in a new Petri dish with a lid for further examination.

### Extraction of microplastics in river water

The water samples (50 mL) were vacuum filtered sequentially with the aid of a suction pump and cellulose base filter paper. After filtration the samples were counted and identified. The particles on the filter paper were dried, picked and counted using the AmScope Trinocular Stereo Zoom Microscope SM-1TY-144-18M3 with Digital Camera 18.0MP stereo microscope. The number of particles were recorded as particles per 50 ml [23].

### Extraction of microplastics in sediment

Dried sediments (1 kg) were weighed and extracted using saturated NaCl solution with the aid of an orbital shaker at 300 rpm for 15 mins thrice. The combined extract for each sample was filtered using a 1 mm stainless steel sieve to which a filter paper had been attached. The retained residue was rinsed with distilled water, dried and counted for the number of particles using a AmScope Trinocular Stereo Zoom Microscope SM-1TY-144-18M3 with Digital Camera 18.0 MP.

### Observation and identification of MPs

The particles were observed under a AmScope Trinocular Stereo Zoom Microscope SM-1TY-144-18M3 with Digital Camera 18.0 MP to confirm they were plastics and counted. The counted MPs were categorized in terms of shape (fiber, fragment and film), color (black, blue, red, white, yellow, green and purple) and size. MPs were measured using a calibrated scaled eyepiece lens [24] and divided into six classes: ≤ 0.5, 0.5–1, 1–2, 2–3, 3–4 and 4–5 mm. Pictures of the particles were taken by digital camera. All suspected MPs identified during sample preparation were subjected to and further analysed using attenuated total reflectance-Fourier transform infrared spectroscopy (ATR-FTIR) for characterization of the MPs. A Nicolette Nexus 470 Attenuated total reflection ATR-FTIR by Therm, USA using an Omic software was used for the identification confirmation, characterization and identification of the MPs. The spectral range for spectrum in this study was in 650–4000 cm^−1^ region and 4 cm region and 4 cm^−1^ resolution. Each spectrum was collected from 32 scans in the Transmittance mode. Triplicate measurements were made and the mean values were used. In order to obtain the FTIR spectra, the transmittance values were plotted (y-axis) as a function of wave number (x-axis), and compared with the library spectra to know the spectra close to the one obtained. The spectra were corrected for error by running a background air spectrum frequently in the run [25]. This comprehensive approach ensured that each suspected microplastic particle within the sample set underwent ATR-FTIR analysis for accurate characterization and identification.

### Exposure assessment modelling

Oral intake might be an individual risk route associated with human exposure to microplastic contamination of fish and water samples. Consequently, the estimated daily intake (EDI) attributable to exposure to MPs as a consequence of consuming contaminated fish and water was calculated using the equation (1) [26].


(1) EDIq= (Pi × Ri)/ Bw

where, EDIq: estimated daily intake of MPs based on quantity (EDIq) through consumption of fish and water from the rivers (MPs particles/g-day or MPs particles/mL-day); Pi: average quantity of the MPs in fish (MP particles/g) or water (MP particles/L) samples; RI: ingestion rate in Nigeria; 20.8 g of fish per day and for water; 2.2 L or 2200 mL per day for adults and 1.8 L or 1800 mL per day for children; BW: average body weight (70000 g for adults; 15000 g for children) as described in earlier reports [26].

### Statistical analysis

Data obtained were presented as mean ± SDV (standard deviation) while percentages and frequency of microplastic occurrence in species and entire sampled population were also performed using OriginLab Pro (Origin software, USA). Principal component analysis (PCA) and Hierarchical Cluster Analysis (HCA) was performed to determine the source of MPs and relationship between rivers while correlation analysis was used for the different matrices using Originlab Pro.

## Results and Discussion

### MP abundances in fishes, water and sediments from the different rivers

Globally, the ingestion of MP (MPs) by fishes is a well-documented phenomenon, making them valuable bioindicators for assessing plastic pollution. The gastrointestinal tract and gills of fishes are major sites for MPs accumulation, as these organs readily absorb particles from the environment with minimal barriers. While the exact source of MP in fish can vary, it is often attributed to contamination from river water and sediments. In our study, we found MPs in 100 % of the sampled fishes, water, and sediments across all studied rivers. This alarming finding underscores the urgent need for policy makers to address the proliferation of MP in these areas. Similar results were reported by Ogbomida et al. [17], who found a 100% prevalence of MP in fishes, water, and sediments from the Ikpoba Rivers of Edo State, Nigeria. Dada and Bello [11], found 100 % MPs in fishes from coastal urban lagoon in Lagos, Nigeria. In contrast, earlier study by Adeogun et al. [12] reported a prevalence of 69.7% of MPs in the stomachs of commercial fish collected from the Eleyele municipal water supply lake in Nigeria.

**Figure 2.**
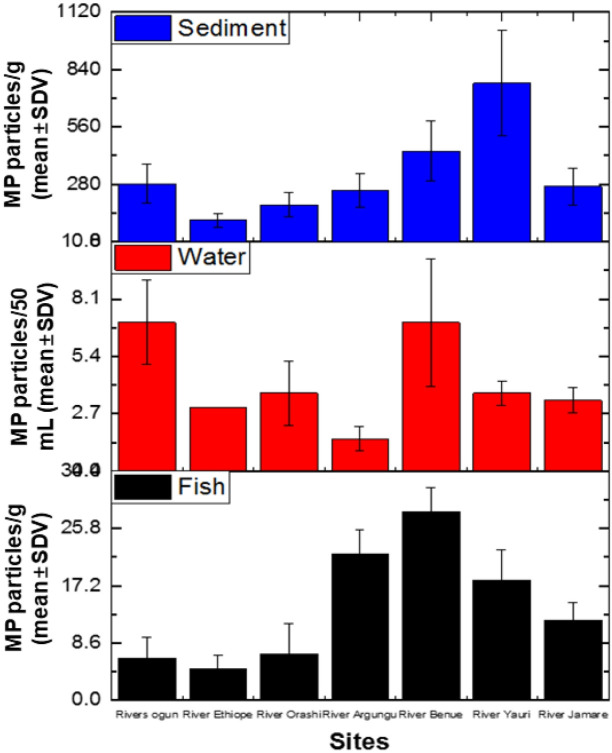
Microplastics abundance in fish, water and sediments samples from the different rivers. Bars indicates standard deviations (SDV) (n = 3).

The mean distribution of MPs in fishes from different rivers followed a pattern, with the highest concentrations found in the River Benue (28.33 ± 3.51 MP particles/g), followed by River Argungu (22.00 ± 3.61 MP particles/g), River Yauri (18.00 ± 4.58 MP particles/g), River Jamare (12.00 ± 2.65 MP particles/g), River Orashi (7.00 ± 4.58 MP particles/g), River Ogun (6.33 ± 3.06 MP particles/g), and River Ethiope (4.67 ± 2.08 MP particles/g) (Figure 2). This suggests that fishes in River Benue are more exposed to MPs, which could be due to higher pollution levels in this river compared to others. This pattern could be influenced by factors such as plastic waste disposal practices, water flow rates, and environmental conditions. Statistical analysis using ANOVA revealed significant differences between River Argungu, River Benue, and River Yauri compared to the other rivers (p < 0.05). However, there were no significant differences (p = 0.47) in MPs concentrations between Rivers Ogun, Ethiope, and Orashi. The concentrations of MPs in fish reported in our study were higher than those reported in previous studies in other rivers in Nigeria. For example, Ilechukwu et al. [27] reported an average of 3.87 ± 5.97 particles per fish in samples collected from the New Calabar River. Similarly, Ogbomida et al. [17] found a maximum average of 7.33 ± 2.08 MP particles in fishes, water, and sediments from the Ikpoba Rivers of Edo State, comparable to the results obtained for River Orashi, River Ogun, and River Ethiope. The higher concentrations of MPs reported in our study could be attributed to the greater presence of plastic litter in these rivers. Previous research has shown a correlation between higher concentrations of marine litter and higher concentrations of MPs [3]. In the water samples, the trend of microplastic (MP) concentrations across different rivers was observed as follows: River Ogun (7.00 ± 2.00 MP particles/ 50 mL) / River Benue (7.00 ± 3.00 MP particles/ 50 mL) > River Orashi (3.67 ± 1.53 MP particles/ 50 mL) > River Yauri (3.67 ± 0.58 MP particles/ 50 mL) > River Jamare (3.33 ± 0.58 MP particles/ 50 mL) > River Argungu (1.53 ± 0.58 MP particles/ 50 mL). This indicates that River Ogun and River Benue had the highest MP concentrations per 50 mL of water, followed by River Orashi, River Yauri, River Jamare, and finally, River Argungu, which had the lowest concentration. In contrast, in the sediment samples, the trend of MP concentrations across different rivers was observed as follows: River Yauri (773 ± 257.67 MP particles/g) > River Benue (441 ± 147 MP particles/g) > River Ogun (283 ± 94.33 MP particles/g) > River Jamare (270 ± 90 MP particles/g) > River Argungu (248.00 ± 82.67 MP particles/g) > River Orashi (180 ± 60 MP particles/g) > River Ethiope (104 ± 34.67 MP particles/g). This indicates that River Yauri had the highest MP concentration per gram of sediment, followed by River Benue, River Ogun, River Jamare, River Argungu, River Orashi, and finally, River Ethiope, which had the lowest concentration. Similar high concentrations of MPs have been reported elsewhere in Nigeria, as noted by Olarinmoye et al. [28] and Dada and Bello [11]. These concentrations have been attributed to fishing activities and the dumping of waste (including human, animal, and solid waste) directly into the rivers. The presence of MPs in such high concentrations highlights the urgent need for effective waste management strategies and environmental conservation efforts to reduce plastic pollution in Nigerian rivers.

### Characteristics of MPs in fishes, water and sediments from the different rivers

#### Shape

**Figure 3.**
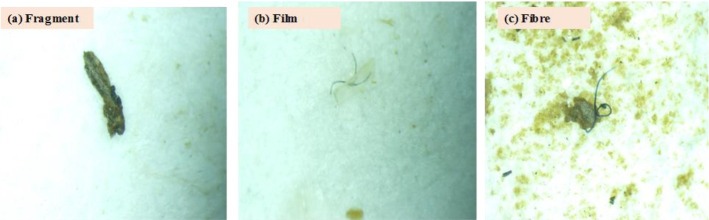
Detected shapes of MPs in fish, water and sediment from the rivers.

**Figure 4.**
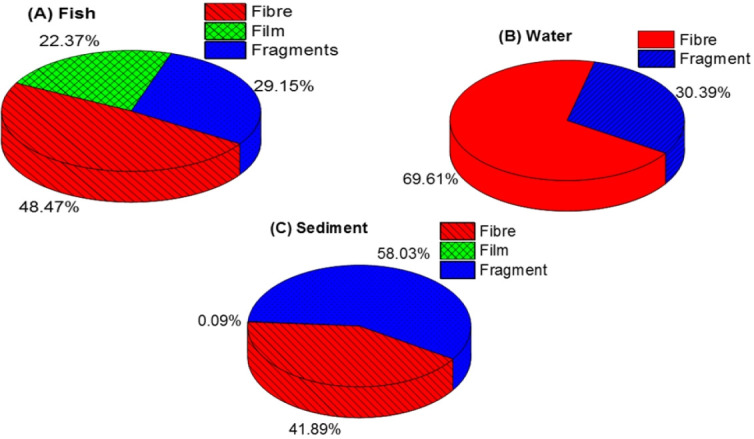
Distribution of MPs based on shapes in fish (A), water (B) and sediment (C) from the rivers.

The microscope analysis revealed three primary shapes of MP: fragments, fibre, and films (Figure 3). Fragment images displayed rough surfaces or jagged edges with cracks, suggesting they were broken off from larger plastics. Fibre shapes appeared thread-like or as long, narrow marks, longer than they were wide. Films exhibited a thin, sheet-like shape. The distribution of MPs based on different shapes is detailed in Table S1 (supplementary data) and illustrated in Figure 4. Generally, both water and fish samples exhibited high abundance of fibers (Figure 4A and 4B). Among fish samples, fibers constituted the highest proportion at 48.47 %, followed by fragments at 29.15 %, and films at 22.37 % (Figure 4A). In water samples, fibers were predominant at 69.61 % compared to fragments at 30.39 % (Figure 4B). In sediment samples, however, the distribution of MPs based on shapes differed, with fragments being the most prevalent at 58.03%, followed by fibers at 41.89 %, and films at a minimal 0.09 % (Figure 4C). Interestingly, films were only detected in sediment samples from Rivers Ogun. River Argungu, on the other hand, exhibited a high abundance of fibers relative to fragments in the samples collected from that site (Table S1).

#### Color

The color composition of MPs in the fishes, water and sediments samples are presented in Figure S1 and S2, (supplementary data), providing insights into the types of plastics present in these environments. In fish samples, the distribution of colors was as follows: Black (32 %) > Brown (21 %) > Blue (20 %) > Cream/Colorless (14 %) > White (6 %) > Red (4 %) > Purple (3 %). This distribution suggests a variety of plastic sources, as different colors are sometimes associated with different types of plastics. Similar color compositions have been reported in other studies of fish samples [29-31], indicating a consistent pattern of plastic pollution in aquatic organisms. In water samples, the color distribution was different, with Blue (40 %) being the most prevalent, followed by Black (32 %), Brown (23 %), Red (4 %), and Orange (1 %). This distribution may reflect the types of plastics commonly found in water bodies, possibly originating from various sources such as packaging materials, textiles, and fishing gear. In sediment samples, the color composition differed again, with Brown (43 %) being the most common color, followed by Black (41 %), Blue (7 %), White (3 %), Red (2 %), Cream/Colorless (2 %), Orange (1%), and Green (< 1 %). The high proportion of Brown and Black plastics in sediments suggests a significant presence of weathered plastics, which may have been present in the environment for a long time.

### Composition

The chemical composition of MP in fish, water, and sediment samples from different rivers was identified using Fourier-transform infrared spectroscopy (FTIR). The MP types identified included polyurethane (PUR), polycarbonate (PC), polyvinyl chloride (PVC), polyamide/nylon (PA/Nylon), polyethylene terephthalate (PET), polyester (PES), polyethylene (PE), polystyrene (PS), and poly (vinyl alcohol) (PVA) (Figure 6). PS and PVA were not detected in the fish samples.

**Figure 5.**
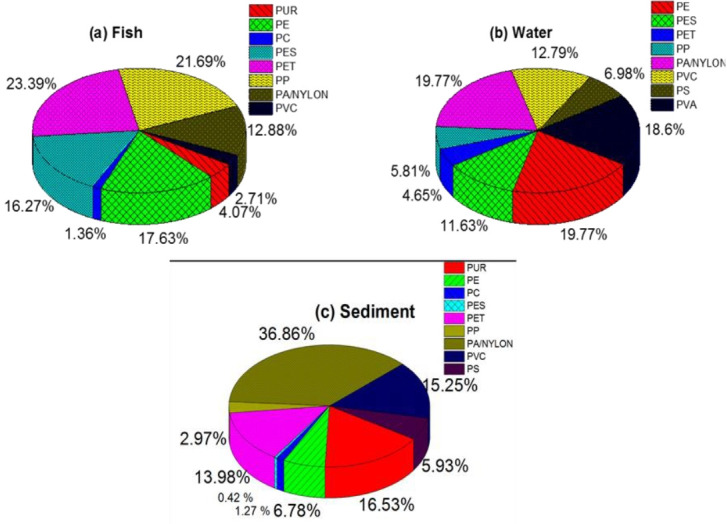
Distribution of MPs by composition category registered in fish samples from the different river.

In fish samples, the distribution of MPs revealed a diverse composition, with different types of plastics present in varying proportions. The most abundant MPs in the fish samples were PET (23.39 %), PP (21.69 %), PE (17.63 %), PES (16.27 %), PUR (4.07 %), PA/Nylon (2.71 %), and PC (1.36 %) (Figure 5a). Comparing these findings with other studies, Pappoe et al. [32] reported the presence of PE, PVA, and PA as the predominant polymer materials found in fishes from the Gulf of Guinea, Ghana. They accounted for 62.70 %, 30.95 %, and 6.35 %, respectively. The distribution of MPs in water samples from the studied rivers showed a varied composition, with different types of plastics present in different proportions. The most abundant MPs in the water samples were PE/PA/Nylon (19.77 %), PVA (18.6 %), PVC (12.79 %), PES (11.63 %), PS (6.98 %), PP (5.81 %), and PET (4.65 %) (Figure 5b). Comparing these findings with other studies in Nigeria, Enyoh et al. [3] conducted a study in South Eastern Nigeria, focusing on rivers Obiaraedu, Nwangele, Okumpi, Ogbajarajara, and Onuezuze. They found that PET (29 %), PE (22%), and PVC (16 %) were the most distributed MPs in these rivers. This suggests some consistency in the types of plastics found in water samples from different locations within Nigeria, with PET and PE being among the most common. Another study by Oni et al. [33] focused on ten sections of OX-Bow Lake Yenagoa, Nigeria, during two different seasons - Dry and Rainy. They found that PETs and Plasticized PVC were predominant, accounting for 72.63 % of the MPs found in the dry season, while PVC accounted for 81.5% of MPs in the rainy season.

**Figure 6.**
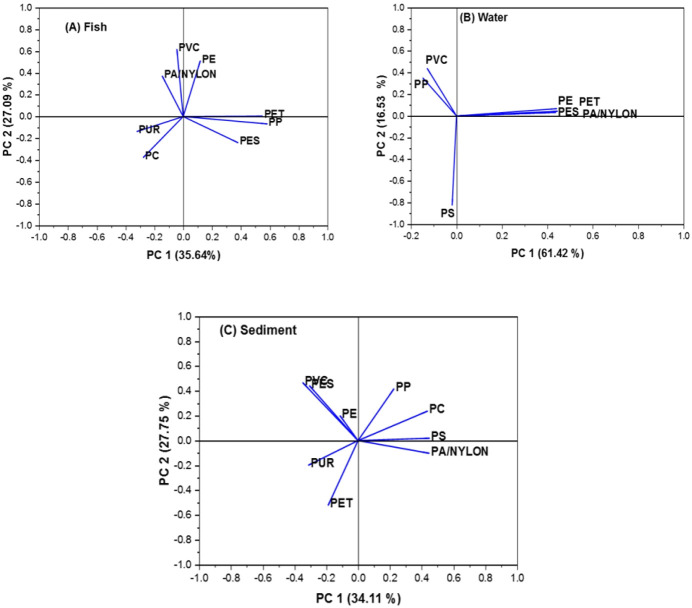
PCA plots for MPs in (a) fish (b) water and (c) sediment.

These studies collectively underscore the widespread presence of MPs in Nigerian water bodies, with PET, PE, and PVC being among the most commonly found types. In sediment samples, the distribution of MPs showed a varied composition, with different types of plastics present in different proportions. The most abundant MPs in the sediment samples were PA/Nylon (36.86 %), PUR (16.53 %), PVC (15.25 %), PET (13.98 %), PE (6.78 %), PS (5.93 %), PP (2.97 %), PC (1.28 %), and PES (0.42 %) (Figure 6c).

Comparing these findings with other studies, Ilechukwu et al. [28] reported the presence of PS, PP, and PE in the surface sediment from a lagoon bordering the urban agglomeration of Lagos, Southwest Nigeria. This suggests a similar trend in the types of plastics found in sediment samples from different locations within Nigeria, with PS, PP, and PE being among the most common. Oni et al. [33]. conducted a study on sediment samples from OX-Bow Lake Yenagoa, Nigeria, during two different seasons - Dry and Rainy. They found that PET and PVC were the predominant MPs, accounting for 10.9% in sediment samples in the dry season, while LDPE (4.2 %) was predominant in the rainy season. These studies collectively underscore the widespread presence of MPs in Nigerian sediment samples, with PA/Nylon, PUR, PVC, and PET being among the most commonly found types.

These results indicate variations in the types and distribution of MPs among the different environmental compartments. For example, fish samples showed a higher proportion of PET and PP, which are commonly used in packaging and textiles. Water samples exhibited a significant presence of PE/PA/Nylon, possibly from industrial or wastewater sources. Sediment samples, on the other hand, had a higher content of PA/Nylon, which could originate from various sources such as textiles and fishing gear. The presence of different types of MPs in samples highlights the extent of plastic pollution in aquatic environments and the potential risks to marine life and human health.

### Source apportionment and relationship between rivers

In order to determine the source or sources of MPs in the fish, water, and sediment samples, principal component analysis, or PCA, was carried out. PCA is a data dimension reduction technique that explains the majority of the variation in the data using a small number of independent variables (referred to as "principal components") [34]. The method employs an orthogonal transformation strategy to obtain the first principle component that explains the most variation in theinal data. The next stage involves identifying each component that comes after by requiring it to be orthogonal to every component that came before it. In most cases, eigenvalue decomposition is used as a component of a matrix operation. The eigenvectors with the greatest eigenvalue ( > 1) make up the majority of the data set.

In Figure 6, the PCA findings are displayed. The analysis of MPs in fish samples identified three principal components (PCs) that collectively explained a significant portion of the variance in plastic types. PC 1 accounted for 35.64 % of the variance and included plastics such as PET (polyethylene terephthalate), PP (polypropylene), and PES (polyester). These plastics are commonly used in packaging materials, textiles, and other consumer products. PC 2 explained 27.09 % of the variance and contained plastics like PE (polyethylene), PVC (polyvinyl chloride), and PA/NYLON (polyamide/nylon). PE is widely used in plastic bags and bottles, while PVC is used in construction materials and medical devices. PA/NYLON is often found in textiles and engineering plastics. PC 3 explained 20.42 % of the variance and included PC (polycarbonate) and PUR (polyurethane). PC is used in electronics, optical media, and medical devices, while PUR is used in foam products, coatings, and adhesives. The identification of these principal components can help inform strategies for reducing plastic pollution in aquatic environments.

The analysis of MPs in river water samples revealed three principal components (PCs). PC1 explained 61.42% of the variance and included plastics like PE, PET, PES, and PA/Nylon (Figure 6). PC2 explained 16.53 % of the variance and contained PVC and PP, while PS was exclusively found in PC3, explaining 15.06 % of the variance. In river sediment samples, MPs were also extracted into three principal components. PC1 explained 34.11 % of the variance, PC2 explained 27.75 %, and PC3 explained 21.02 %. The plot in rotated space showed that PP, PC, PS, and PA/Nylon were grouped in PC1; PVC, PES, and PE in PC2; and PUR and PET in PC3. The distribution of plastics in the river water and sediment samples suggests different sources and pathways of contamination. PE, PET, PES, and PA/Nylon found in PC1 of the water samples are commonly used in packaging and textiles, indicating potential sources such as urban runoff, industrial discharge, or improper waste disposal. PVC and PP in PC2 of the water samples are also used in packaging and consumer products, suggesting similar contamination sources. PS, found exclusively in PC3 of the water samples, is used in packaging, insulation, and other applications, indicating a different source or pathway of contamination. In the sediment samples, the distribution of plastics in the three principal components suggests a similar pattern (Figure 6). Plastics like PP, PC, PS, and PA/Nylon in PC1 are commonly used in various consumer products and industrial applications. PVC, PES, and PE in PC2 are also used in packaging and textiles, while PUR and PET in PC3 are used in consumer products and textiles.

The source of MPs in the different matrices were evaluated by correlation analysis. The results (not shown) indicated a significant positive correlation between fish and water (r > 0.5), suggesting that the presence of MPs in fish is related to the MPs present in the water. Similarly, there was a significant positive correlation between sediments and fish (r > 0.5), indicating that the presence of MPs in fish is also related to the MPs present in sediments. However, the correlation between water and sediments was not significant (r = 0.074), suggesting that the presence of MPs in water may not be strongly related to the MPs present in sediments. These findings imply that fish may be ingesting MPs from both water and sediments, but the correlation between water and sediments is not as strong, indicating that the sources of MPs in these matrices may be different or influenced by other factors.

The Hierarchical Cluster Analysis (HCA) conducted in this study aimed to explore the relationship between rivers based on the distribution of MPs found in fish, water, and sediment samples. HCA is an unsupervised learning technique that sorts units into groups (clusters) based on their similarity, with items in the same group being more similar to each other than to those in other groups [26].

**Figure 7.**
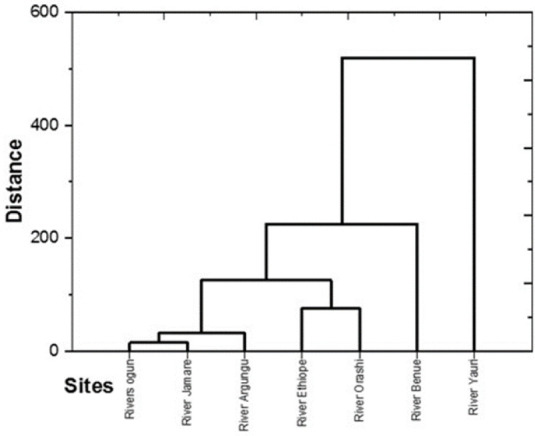
Dendogram for Rivers based on the MPs recorded in fish, water and sediment samples.

The dendrogram resulting from the HCA, as shown in Figure 7, indicates how the rivers are clustered based on their similarity in MP distribution. Rivers Ogun, Jamare, and Argungu are clustered together, suggesting a similarity in the types and abundance of MPs found in samples from these rivers. Similarly, Rivers Ethiope and Orashi are clustered together, while River Benue and Yanuri are in separate clusters. The geographical distribution of these rivers, as shown in Figure 1, provides additional context to the clustering results. Rivers Jamare and Argungu are located in the far north of Nigeria, while Orashi and Ethiope are in the southeast. River Benue and Yanuri are uniquely located on the map. This geographical distribution likely influences the distribution of MPs in the fish, water, and sediment samples, as different rivers may be exposed to similar and varying levels of anthropogenic activities and pollution sources. These HCA results suggest that the geographical location and possibly the associated human activities are important factors influencing the distribution of MPs in the studied rivers.

### MPs exposure assessment modelling

**Figure 8.**
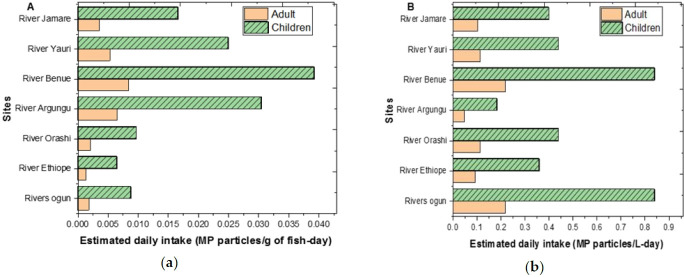
Quantity based estimated daily intake from ingestion of fish (a) and water (b) from the different rivers.

The concept of a risk model, as described, involves using statistical methods to estimate the probability of an individual experiencing an adverse outcome within a specified time frame. In the context of MPs exposure, such models can help assess the likelihood of harm from consuming contaminated fish and water. Figure 8 illustrates the estimated daily intake (EDI) of MPs from consuming fish and river water samples, considering both adults and children. The results suggest that the EDIs are generally low, indicating that daily consumption of the samples is not likely to be harmful. In comparison with Pham [35] for fish sauce in South Korea, a 1.1 g of MPs are consumed per week (0.15 g-day), which is higher than those recorded in this study. However, it's important to note that even at low levels, MPs can have negative effects on human health, as indicated by meta-regression assessments showing impacts on cell viability and cytokine release [36]. This study found that children tend to consume more MPs than adults, a trend consistent with previous reports [37]. This finding underscores the importance of considering age-related differences in exposure when assessing the health risks associated with MPs. The identification of River Benue (Figure 8) as having the highest EDI for both fish and water samples highlights the need for targeted interventions in areas with higher potential exposure.

## Conclusions

This study provides a comprehensive assessment of MPs in fishes, sediment, and water from inland rivers across Nigeria's six geopolitical zones. The findings reveal a widespread presence of MPs in all sampled compartments, indicating significant plastic pollution in these aquatic environments. The presence of MPs in 100 % of the sampled compartments highlights the urgent need for policymakers to address plastic pollution in aquatic environments. The distribution of MPs showed that River Benue had the highest concentrations in fishes, Rivers Ogun and Benue had the highest concentrations in water samples while Rivers Yauri and Benue in sediment. The shape distribution of MPs varied among samples, with fibers being predominant in both water and fish samples, and fragments being more prevalent in sediment samples. The color composition of MPs also varied, indicating a diverse range of plastic sources. The distribution and composition of MPs vary among the different rivers and environmental compartments, suggesting multiple sources and pathways of contamination. The identification of PET, PP, PE, and PA/Nylon as the most abundant plastic types underscores the importance of addressing plastic waste from packaging and textiles. The results of the principal component analysis (PCA) and hierarchical cluster analysis (HCA) provide insights into the sources and relationships between rivers based on MP distribution. Overall, this study highlights the urgent need for effective waste management strategies and environmental conservation efforts to mitigate plastic pollution in Nigerian rivers.

Recommendations: Based on the findings of this study, the following recommendations are proposed:

1. Implement strict regulations and policies to reduce the disposal of single-use plastics, especially PET, PP, PE, and PA/Nylon, in inland waters in Nigeria.

2. Conduct specific further research to investigate the sources and pathways of MPs in inland rivers, including the role of urban runoff, industrial discharge, and agricultural activities.

3. Collaborate with stakeholders, including government agencies such as Lagos Waste Management Authority (LAWMA) etc to develop and implement effective strategies for reducing plastic pollution and protecting aquatic ecosystems in Nigeria.
